# Pigs Lacking the Scavenger Receptor Cysteine-Rich Domain 5 of CD163 Are Resistant to Porcine Reproductive and Respiratory Syndrome Virus 1 Infection

**DOI:** 10.1128/JVI.00415-18

**Published:** 2018-07-31

**Authors:** Christine Burkard, Tanja Opriessnig, Alan J. Mileham, Tomasz Stadejek, Tahar Ait-Ali, Simon G. Lillico, C. Bruce A. Whitelaw, Alan L. Archibald

**Affiliations:** a The Roslyn Institute, Royal (Dick) School of Veterinary Studies, University of Edinburgh, Easter Bush, Midlothian, United Kingdom; b Department of Veterinary Diagnostic and Production Animal Medicine, College of Veterinary Medicine, Iowa State University, Ames, Iowa, USA; c Genus plc, DeForest, Wisconsin, USA; d Warsaw University of Life Sciences, Faculty of Veterinary Medicine, Department of Pathology and Veterinary Diagnostics, Warsaw, Poland; Loyola University Medical Center

**Keywords:** CD163, CRISPR/Cas9, PRRSV, arterivirus, exon deletion, genome editing, nidovirus, porcine reproductive and respiratory syndrome virus, resistance

## Abstract

Porcine reproductive and respiratory syndrome virus (PRRSV) has a narrow host cell tropism, limited to cells of the monocyte/macrophage lineage. CD163 protein is expressed at high levels on the surface of specific macrophage types, and a soluble form is circulating in blood. CD163 has been described as a fusion receptor for PRRSV, with the scavenger receptor cysteine-rich domain 5 (SRCR5) region having been shown to be the interaction site for the virus. As reported previously, we have generated pigs in which exon 7 of the CD163 gene has been deleted using CRISPR/Cas9 editing in pig zygotes. These pigs express CD163 protein lacking SRCR5 (ΔSRCR5 CD163) and show no adverse effects when maintained under standard husbandry conditions. Not only was ΔSRCR5 CD163 detected on the surface of macrophage subsets, but the secreted, soluble protein can also be detected in the serum of the edited pigs, as shown here by a porcine soluble CD163-specific enzyme-linked immunosorbent assay (ELISA). Previous results showed that primary macrophage cells from ΔSRCR5 CD163 animals are resistant to PRRSV-1 subtype 1, 2, and 3 as well as PRRSV-2 infection *in vitro*. Here, ΔSRCR5 pigs were challenged with a highly virulent PRRSV-1 subtype 2 strain. In contrast to the wild-type control group, ΔSRCR5 pigs showed no signs of infection and no viremia or antibody response indicative of a productive infection. Histopathological analysis of lung and lymph node tissue showed no presence of virus-replicating cells in either tissue. This shows that ΔSRCR5 pigs are fully resistant to infection by the virus.

**IMPORTANCE** Porcine reproductive and respiratory syndrome (PRRS) virus (PRRSV) is the etiological agent of PRRS, causing late-term abortions, stillbirths, and respiratory disease in pigs, incurring major economic losses to the worldwide pig industry. The virus is highly mutagenic and can be divided into two species, PRRSV-1 and PRRSV-2, each containing several subtypes. Current control strategies mainly involve biosecurity measures, depopulation, and vaccination. Vaccines are at best only partially protective against infection with heterologous subtypes and sublineages, and modified live vaccines have frequently been reported to revert to virulence. Here, we demonstrate that a genetic-control approach results in complete resistance to PRRSV infection *in vivo*. CD163 is edited so as to remove the viral interaction domain while maintaining protein expression and biological function, averting any potential adverse effect associated with protein knockout. This research demonstrates a genetic-control approach with potential benefits in animal welfare as well as to the pork industry.

## Abstract (precis)

Porcine reproductive and respiratory syndrome (PRRS) virus (PRRSV) is the etiological agent of PRRS, causing late-term abortions, stillbirths, and respiratory disease in pigs, incurring major economic losses to the worldwide pig industry. The virus is highly mutagenic and can be divided into two species, PRRSV-1 and PRRSV-2, each containing several subtypes. Current control strategies mainly involve biosecurity measures, depopulation, and vaccination. Vaccines are at best only partially protective against infection with heterologous subtypes and sublineages, and modified live vaccines have frequently been reported to revert to virulence. Here, we demonstrate that a genetic-control approach results in complete resistance to PRRSV infection *in vivo*. CD163 is edited so as to remove the viral interaction domain while maintaining protein expression and biological function, averting any potential adverse effect associated with protein knockout. This research demonstrates a genetic-control approach with potential benefits in animal welfare as well as to the pork industry.

## INTRODUCTION

Porcine reproductive and respiratory syndrome (PRRS) is arguably the most economically important infectious disease affecting pigs worldwide. The causative agent of PRRS is PRRS virus (PRRSV), a member of the Arteriviridae family and the order *Nidovirales*. Infected pigs of all ages may present with symptoms involving inappetence, fever, lethargy, and respiratory distress. However, the most devastating effects of PRRSV infection are observed in young piglets and pregnant sows. In pregnant sows, full abortions or death and mummification of fetuses *in utero* are observed, and live-born piglets from an antenatal infection are often weak and display severe respiratory symptoms (1–3). Piglets infected with PRRSV in early life can show diarrhea and, more commonly, severe respiratory distress due to active PRRSV replication in pulmonary macrophages and subsequent damage in lung tissues (4). Due to the reduction or loss of pregnancies, death in young piglets, and decreased growth rates in all PRRSV-infected pigs, it is estimated that the economic impact of PRRSV to pork producers in the United States alone is more than $650 million annually (5, 6).

There are two different species of PRRSV with distinct geographic distributions: PRRSV-1 is found primarily in Europe and Asia, overlapping the range of PRRSV-2, which is found in Asia and the Americas. PRRSV-1 can be further divided into at least three subtypes, currently based on open reading frame 7 (ORF7) sequences and geographical distribution, with subtype 1 being pan-European and subtypes 2 and 3 currently being limited to eastern Europe (7).

PRRSV has a very narrow host cell range, infecting only specific subsets of porcine macrophages (8–10). Entry of PRRSV into macrophages has been shown to occur via pH-dependent, receptor-mediated endocytosis (11, 12). Various attachment factors and receptors have been indicated to be involved in the PRRSV entry process (reviewed in reference 13). However, only the scavenger receptor CD163, also known as a hemoglobin (Hb)-haptoglobin (Hp) scavenger receptor or p155, has been confirmed to be an essential fusion receptor *in vitro* and *in vivo* (14–16). CD163 is expressed on specific subtypes of macrophages. The extracellular portion of CD163 forms a pearl-on-a-string structure of nine scavenger receptor cysteine-rich (SRCR) domains and is anchored by a single transmembrane segment and a short cytoplasmic domain (17). A proteolytically cleaved, soluble form of the protein ectodomain is found in the bloodstream and is involved in the inflammation and ischemic repair response (18, 19). Transmembrane anchoring and interaction with SRCR domain 5 (SRCR5) of CD163 were found to be essential for successful infection with PRRSV (20, 21). CD163 has a variety of biological functions, including mediating systemic inflammation and the removal of hemoglobin from blood plasma (reviewed in references 21 and 22). Overexpression of CD163 renders nonsusceptible cells permissive to PRRSV infection (20), and it was found that CD163 does not mediate internalization but is crucial for fusion (16).

Recent *in vivo* challenge experiments of pigs in which both copies of the CD163 gene had been knocked out using gene-editing technology confirmed that CD163 is required for infection by PRRSV-2 and highly pathogenic PRRSV-2 (HP-PRRSV) (14, 23). Gene-editing technology has also been used to generate pigs in which the CD163 SRCR5-encoding sequence has been replaced with a sequence encoding human CD163L1 SRCR8 (24), in effect replicating *in vivo* the previous *in vitro* domain-swapping experiment of Van Gorp and colleagues (25). This attempt to maintain CD163 function rendered pigs and macrophages resistant to PRRSV-1 but not PRRSV-2 infection (24), making this strategy ineffective in combating both PRRSV species. CD163 has important biological functions, and the complete knockout could have a negative physiological impact on the animal, particularly with respect to the inflammation response and/or infection by other pathogens. Interestingly, whereas all the other eight SRCR domains have been shown to be involved in different biological functions, no specific role has been associated with SRCR5, other than in PRRSV infection (21). Therefore, we generated pigs lacking SRCR5 by the deletion of exon 7 of *CD163* using CRISPR/Cas9 editing and showed that macrophages from these pigs were resistant to both PRRSV-1 and PRRSV-2 infection *in vitro* (15). The aim of the experiments described here was to determine whether our *in vitro* results would translate directly *in vivo* by conducting a PRRSV challenge of pigs with a CD163 SRCR5 deletion. Furthermore, we aimed to characterize the biological function of the modified CD163 protein (ΔSRCR5 CD163) as both a soluble and a cell-bound protein.

## RESULTS

### Genome editing in zygotes for ΔSRCR5 CD163 pigs and breeding genotypically uniform F2 pigs.

**FIG 1:**
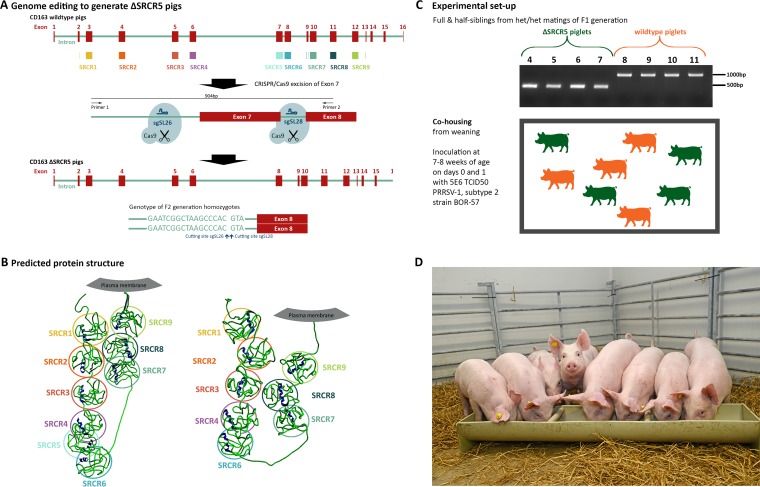
Generation of ΔSRCR5 pigs and experimental setup. (A) Genome editing to generate ΔSRCR5 pigs. Genome-edited founder animals were generated by zygote injection of CRISPR/Cas9 editing reagents using Cas9 mRNA and two guide RNAs, sgSL26 and sgSL28, in combination to generate a deletion of exon 7 in CD163. Animals were bred to generate F1 and F2 generations, focusing on one genotype showing clean religation at the cutting sites of both guide RNAs. Homozygous F2 animals carry this genotype in both alleles (bottom). (B) Structure prediction and expression of ΔSRCR5 in pulmonary alveolar macrophages of F2 animals. Protein structure prediction using RaptorX points toward an intact protein product upon the deletion of SRCR5. (C) Experimental design of the challenge study. Four homozygous (green) and 4 wild-type (orange) siblings from heterozygous/heterozygous mating of the F1 generation animals were cohoused from weaning. Genotypes were confirmed by PCR amplification across exon 7 (see panel A) and by Sanger sequencing. Piglets were cohoused after weaning and after acclimation to the specific-pathogen-free unit for 1 week and throughout the 14-day challenge experiment that was initiated by inoculating each pig intranasally with 5E6 TCID_50_ of PRRSV-1 subtype 2 strain BOR-57 at day 0 and day 1 of the challenge. The piglets were 7 to 8 weeks of age at the start of the acclimation period. (D) Piglets 1 day before the start of the challenge.

Founder-generation (F0) animals carrying a deletion of exon 7 in the CD163 gene, which encodes SRCR5 of the protein, were generated by CRISPR/Cas9 gene editing as previously described (ΔSRCR5 pigs) (15). Briefly, zygotes were microinjected with a combination of Cas9 mRNA and guide RNAs targeting sites flanking exon 7, resulting in double-strand breaks (DSBs) and the deletion of the exon (Fig. 1A). Cross-breeding of heterozygous founder animals and outbreeding with wild-type pigs yielded a first generation composed of both heterozygous and biallelic edited animals (F1 generation) (15). We selected for breeding heterozygous F1 animals displaying a “clean” deletion between the DSBs, i.e., no “on-target” sequence changes beyond the desired deletion region. To generate a cohort with comparable genetic backgrounds, half-sibling heterozygous animals and wild-type animals were bred to yield homozygous ΔSRCR5 animals (Fig. 1A) and wild-type half- and full-sibling animals.

As described above, ΔSRCR5 animals express the ΔSRCR5 CD163 mRNA and protein at levels equivalent to CD163 transcript and protein levels in wild-type siblings. Furthermore, the native-structure ΔSRCR5 CD163 is recognized on the surface of pulmonary alveolar macrophages (PAMs) by a commercial antibody (15). We further analyzed whether template-based protein structure analysis by RaptorX predicted the folding of each subdomain compatible with a functional ΔSRCR5 CD163 protein (26). As demonstrated in Fig. 1B, all subdomains in both full-length and ΔSRCR5 CD163 are predicted to adopt the globular structure and retain the pearl-on-a-string configuration of the native CD163 protein. This supports our findings indicating the proper folding and expression of the ΔSRCR5 protein.

Previously, we have shown that PAMs and *in vitro*-differentiated peripheral blood monocytes (PBMCs) are resistant to infection with both PRRSV-1 and PRRSV-2. Now, we aimed to assess the resistance of these pigs to PRRSV-1 infection *in vivo*. We selected four homozygous ΔSRCR5 F2 animals and four wild-type controls that were cohoused from weaning (Fig. 1C). Blood samples were taken from all eight pigs, and a full blood count was conducted by the diagnostics laboratory at the Royal (Dick) School of Veterinary Sciences, University of Edinburgh. The blood counts of all animals were within reference values, indicating good general health and the absence of infection or inflammation. Furthermore, the hemoglobin levels of all animals were within reference values, indicating normal function of the hemoglobin/haptoglobin-scavenging activity of CD163 (Table 1).

**TABLE 1:** Whole-blood-count results for ΔSRCR5 (animals 4 to 7) and wild-type (animals 8 to 11) piglets at week 6^*a*^

Indicator	Value for animal								Reference value (range)
	4	5	6	7	8	9	10	11	
WBC count (10E9/liter)	22.5	24	14	15.1	12.4	19.6	26.1	14.4	11–22
Neutrophil count (segmented) (10E9/liter)	5.85	4.8	4.62	5.889	4.34	7.252	7.83	4.32	2–15
% neutrophils (segmented)	26	20	33	39	35	37	30	30	20–70
Neutrophil count (nonsegmented) (10E9/liter)	0	0	0	0	0	0	0	0	0–0.8
% neutrophils (nonsegmented)	0	0	0	0	0	0	0	0	0–4
Lymphocyte count (10E9/liter)	15.3	18.72	8.82	8.305	7.564	11.76	16.182	9.36	3.8–16.5
% lymphocytes	68	78	63	55	61	60	62	65	35–75
Monocyte count (10E9/liter)	0.675	0.48	0.42	0.755	0.496	0.588	1.044	0.576	0–1
% monocytes	3	2	3	5	4	3	4	4	0–10
Eosinophil count (10E9/liter)	0.675	0	0	0.151	0	0	1.044	0.144	0–1.5
% eosinophils	3	0	0	1	0	0	4	1	0–15
Basophil count	0	0	0.14	0	0	0	0	0	0–0.5
% basophils	0	0	1	0	0	0	0	0	0–3
RBC count (10E12/liter)	6.03	6.64	6.99	6.58	6.3	6.53	7.52	6.97	5–9
PCV/hematocrit	0.384	0.391	0.383	0.388	0.382	0.39	0.429	0.421	0.36–0.43
Hb level (g/dl)	11.5	11.9	10.9	11.8	11.6	12	13.8	12.3	10–16
MCV (fl)	63.7	58.9	54.8	58.9	60.7	59.8	57.1	60.5	50–62
MCHC (g/dl)	29.9	30.4	28.3	30.5	30.3	30.9	32.1	29.1	30–36
No. of platelets	219	230	605	397	483	519	219	606	120–720
RDW	20.9	23.1	28.9	20.6	21	18	17	22.6	

a WBC, white blood cell; RBC, red blood cell; PCV, packed cell volume; Hb, hemoglobin; MCV, mean corpuscular volume; MCHC, mean corpuscular hemoglobin concentration; RDW, red cell distribution width.

**FIG 2:**
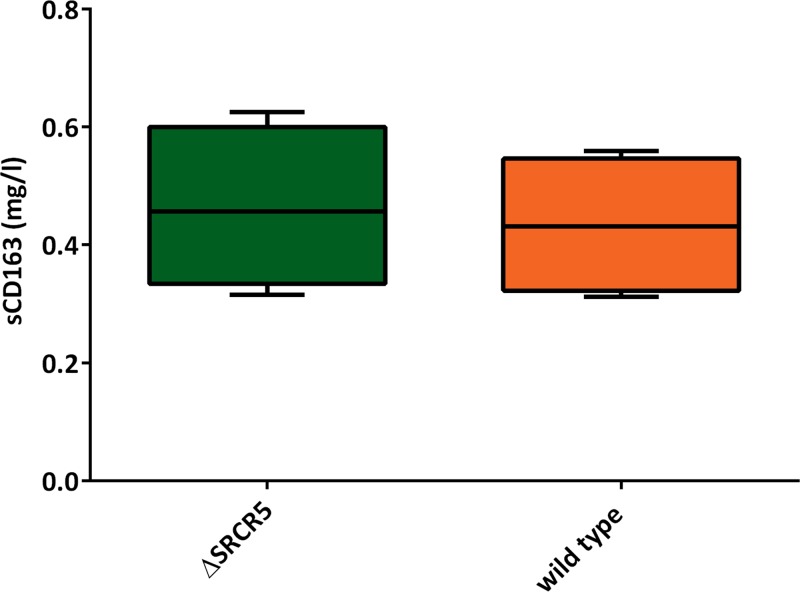
Serum levels of soluble C163. Serum samples collected 2 weeks prior to and on day 0 of the challenge were assessed for the level of sCD163 using a commercial ELISA (*n* = 4 pigs per genotype, serum collected at 2 different time points, assessed in duplicate in 3 replicates). Minima/maxima and 90th percentiles are displayed. Statistical analysis using an unpaired *t* test showed no significant difference.

At 6 weeks of age, a serum sample was collected from all animals prior to movement to a specific-pathogen-free unit (SPFU). The cohort was cohoused for the duration of the experiment and allowed to settle for 1 week prior to the initiation of the challenge. On day 0 of the challenge with PRRSV-1, a second serum sample was taken, and soluble CD163 (sCD163) serum levels were assessed using a commercially available enzyme-linked immunosorbent assay (ELISA) recognizing soluble porcine CD163. Serum CD163 levels were found to be 433.2 ± 69.57 ng/ml in wild-type pigs and 463.5 ± 68.99 ng/ml in ΔSRCR5 pigs (Fig. 2). These results are not significantly different from each other (*P* = 0.7512) and are comparable to sCD163 levels in humans (for example, see reference 27).

### ΔSRCR5 pigs are resistant to PRRSV-1 infection.

At 7 to 8 weeks of age, the pigs were inoculated intranasally with PRRSV-1 subtype 2 strain BOR-57 (28). Eastern European strains are often associated with higher pathogenicity than other PRRSV-1 strains. For BOR-57, we previously observed mild respiratory symptoms, elevated core temperature, extensive lung pathology, and high viral loads in serum. For this study, the strain was selected due to the high viremia and shedding levels expected to occur under study conditions. The experiment was conducted for a period of 14 days following inoculation of each pig on days 0 and 1 with 5E6 50% tissue culture infective doses (TCID_50_), as determined by assessment on PAMs, of the virus isolate used. Rectal temperature, respiration, nasal discharge, coughing, and demeanor were recorded every day, and serum samples were collected on day 0 (prior to challenge) and on days 3, 7, 10, and 14 (prior to euthanasia). Weights were recorded on days 0, 7, and 14 (prior to euthanasia). People assessing the pigs clinically, conducting the challenge, and analyzing pathology were blind to the genotype status of the animals.

**FIG 3:**
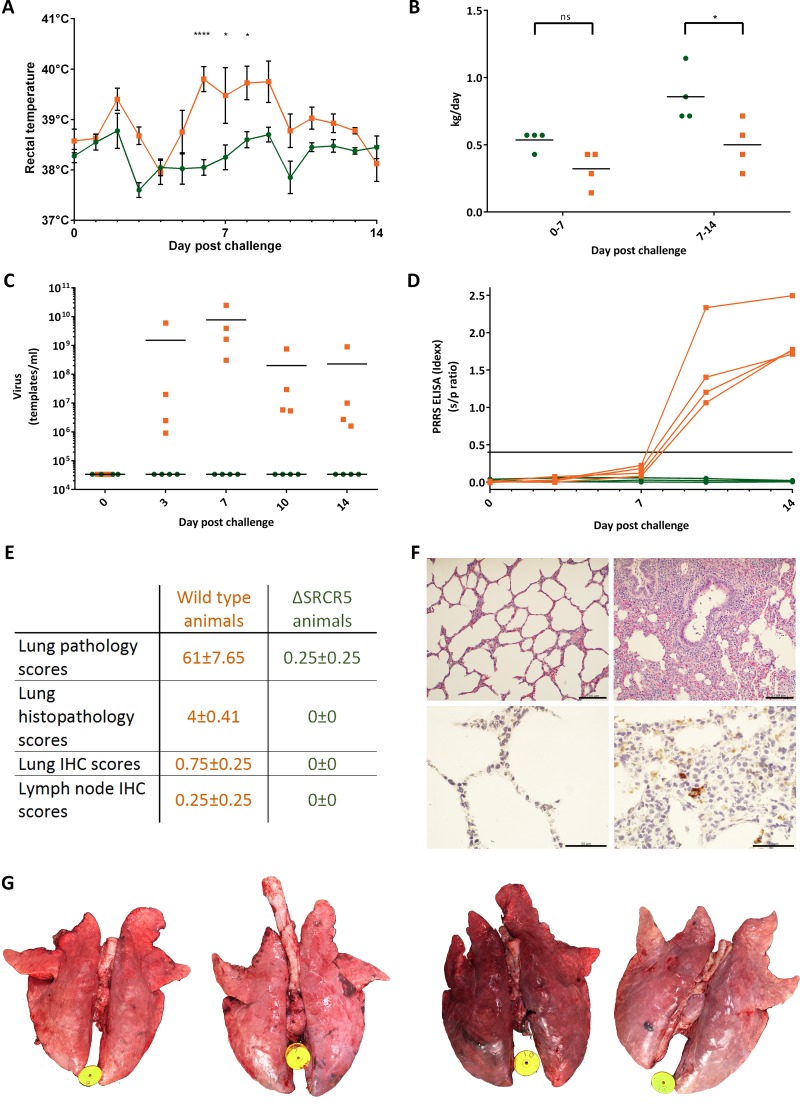
ΔSRCR5 pigs show no clinical signs or pathology of PRRSV-1 infection. (A) Rectal temperatures of ΔSRCR5 (green) and wild-type (orange) piglets during challenge with BOR-57. Rectal temperatures were measured daily during feeding. Error bars represent standard errors of the means (SEM) (*n* = 4). (B) Average daily weight gain based on weight measurements at day 0, 7, and 14 of the challenge. For panels A and B, statistical analysis was performed using two-way ANOVA and Sidak's multiple-comparison test. ns, not significant. (C) Viremia during challenge with BOR-57. Serum samples were collected at days 0, 3, 7, 10, and 14 from the jugular vein using vacuum tubes, and viral RNA was isolated and quantified using RT-qPCR with primers specific to ORF5 of BOR-57. (D) Antibody response to PRRSV-1 during the challenge. Serum samples were analyzed for the presence of PRRSV antibodies using the Idexx PRRSV X3 ELISA, where a value of <0.40 is negative and a value of ≧0.4 is positive. Each data point/line represents data for a single animal, with 4 animals per genotype group. (E) Lung and lymph node pathology, histopathology, and immunohistochemistry (IHC) scores. Lung pathology was assessed in a blind fashion, and a subjective score for the severity of gross lung lesions using an established scoring system was applied (scale, 0 to 100). Lung histopathology sections were scored for the presence and severity of interstitial pneumonia, ranging from 0 to 6 (0, normal; 1, mild multifocal; 2, mild diffuse; 3, moderate multifocal; 4, moderate diffuse; 5, severe multifocal; 6, severe diffuse). Immunohistochemistry staining against PRRSV-N of lung and lymph node sections was scored, ranging from 0 to 3 (0, no signal; 1, low numbers of positive cells; 2, moderate numbers of positive cells; 3, abundant). Numbers represent averages (*n* = 4) ± SEM. (F) Lung histology and immunohistochemistry. (Top) Formalin-fixed, paraffin-embedded, hematoxylin-and-eosin-stained lung sections from necropsy on day 14 postchallenge. (Left) ΔSRCR5 piglets; (right) wild-type piglets. Bar, 100 μm. (Bottom) Formalin-fixed, paraffin-embedded immunohistochemical staining against PRRSV antigen (brown) and hematoxylin counterstain. (Left) ΔSRCR5 piglets; (right) wild-type piglets. The scale bar represents 50 μm. (G) Lung pathology. Shown are lungs from pigs at necropsy at 14 days postchallenge. (Left) Lungs from two ΔSRCR5 pigs; (right) lungs from two wild-type pigs.

The rectal temperatures were significantly elevated on days 6 to 9 of the challenge in the wild-type animals, whereas no fever was observed in the ΔSRCR5 animals (Fig. 3A). The average daily weight gain of the ΔSRCR5 pigs was higher than that of their wild-type counterparts over the entire challenge period and significantly higher over days 7 to 14 (*P* = 0.0465) (Fig. 3B). One wild-type pig showed decreased demeanor on days 7 to 8; no respiratory symptoms or other abnormalities in behavior were observed in any of the other animals during the course of the study, as expected for a PRRSV-1 infection at this age. Viral RNA was isolated from serum, virus levels were quantified by using a DNA fragment template standard, and viral RNA was extracted from virus stocks of known infectivity. Whereas the wild-type pigs showed high viremia, no viral RNA was detected in the serum of ΔSRCR5 pigs (Fig. 3C). The presence of antibodies against PRRSV was assessed using a commercial ELISA able to detect antibodies against all PRRSV-1 subtypes and PRRSV-2. PRRSV antibodies were detected in wild-type pigs from day 7 and present at significant levels (according to the manufacturer's indicated positive threshold of a sample-to-positive (s/p) ratio of >0.4) on days 10 and 14 (Fig. 3D) but were not detected in samples from the ΔSRCR5 pigs collected during the course of the experiment.

During necropsy, lungs were assessed initially, and detailed photographs of the dorsal and ventral sides were taken. Lungs were scored for the presence of lesions. An established scoring system, based on the approximate contribution of each lung section to the complete lung volume, was employed (29). On average, 61% of the lung surface of control pigs was found to be mottled tan with areas of consolidations, compared to 0.25% of lung surfaces in ΔSRCR5 pigs (Fig. 3E and G). Samples of the lungs were fixed in formalin, embedded in paraffin, cut into sections, and stained for further analysis. To assess general lung histology, samples were stained with hematoxylin and eosin. Sections from each pig were assessed for the presence of interstitial pneumonia on a scale of 0 to 6 (0, normal; 1, mild multifocal; 2, mild diffuse; 3, moderate multifocal; 4, moderate diffuse; 5, severe multifocal; 6, severe diffuse). Microscopic lung lesions characterized by multifocal-to-diffuse interstitial pneumonia with type 2 pneumocytes, hypertrophy, and hyperplasia were observed only in wild-type animals (average score, 4) and were not present in lung sections of ΔSRCR5 pigs (Fig. 3E and F, top). The presence of PRRSV antigens was assessed by immunohistochemistry on lung sections and lymph node sections using a mixture of two different antibodies against the PRRSV N protein as described previously (30). PRRSV antigens were detected in 3 out of 4 lung sections and 1 out of 4 lymph node sections of wild-type animals, but no PRRSV antigens were detected in sections from ΔSRCR5 pigs (Fig. 3E and F, bottom).

Overall, no signs of infection were detected in ΔSRCR5 animals despite the high initial inoculation volume and persistent exposure to infected wild-type animals that actively shed virus (the wild-type and edited pigs were cohoused throughout the experiment). This is a clear demonstration that ΔSRCR5 animals are resistant to PRRSV-1 infection, confirming our previous *in vitro* results (15).

### ΔSRCR5 pigs show no cytokine response to PRRSV-1 infection and generally normal cytokine levels.

**FIG 4:**
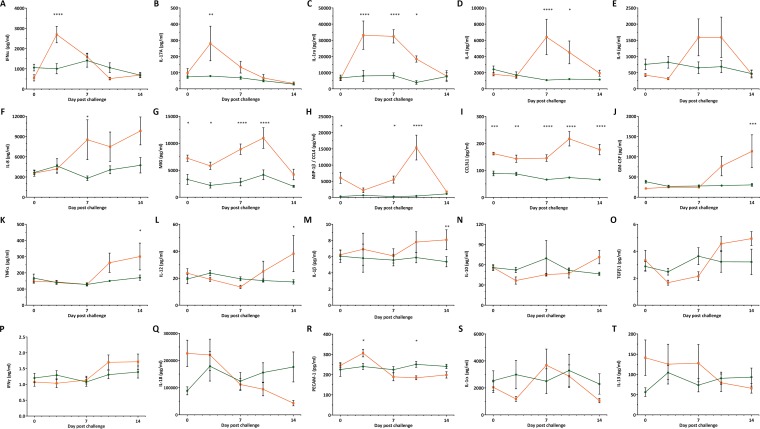
Cytokine response to BOR-57 PRRSV infection. Cytokine levels in serum samples collected prior to challenge on day 0 and on challenge days 3, 7, 10, and 14 were measured using cytokine antibody arrays. (A) Alpha interferon (IFN-α); (B) interleukin-17A (IL-17A); (C) interleukin-1 receptor antagonist (IL-1ra); (D) IL-4; (E) IL-6; (F) IL-8; (G) monokine induced by gamma interferon (MIG/CXCL9); (H) macrophage inflammatory protein 1β (MIP-1β/CCL4); (I) chemokine ligand 3-like 1 (CCL3L1); (J) granulocyte-macrophage colony-stimulating factor (GM-CSF); (K) tumor necrosis factor alpha (TNF-α); (L) IL-12; (M) IL-1β; (N) IL-10; (O) transforming growth factor β1 (TGFβ1); (P) IFN-γ; (Q) IL-18; (R) platelet endothelial cell adhesion molecule 1 (PECAM-1/CD31); (S) IL-1α; (T) IL-13. Error bars represent SEM (duplicates of 4 replicates). Statistical analysis was performed using two-way ANOVA and Sidak's multiple-comparison test. *, *P* ≤ 0.5; **, *P* ≤ 0.01; ***, *P* ≤ 0.001; ****, *P* ≤ 0.0001.

CD163 is involved in the cytokine response to infection and immune stimuli as well as hemoglobin-haptoglobin (Hb-Hp) uptake. Hb-Hp stimulation has been shown to lead to increased levels of interleukin-6 (IL-6) and IL-10 as well as IL-1 and tumor necrosis factor alpha (TNF-α) downregulation (31, 32). SRCR domains 1 to 4 and 6 to 9 contain binding sites for TNF-like weak inducer of apoptosis (TWEAK), an anti-inflammatory cytokine that negatively regulates CD163 expression (33, 34). Stimulation with inflammatory mediators can induce the secretion of soluble CD163 and TNF-α in an ADAM17-mediated manner (35). In order to assess these biological functions in the ΔSRCR5 pigs, we assayed expression levels of key cytokines. Quantitative antibody arrays were used to assess the expression levels of 20 cytokines in serum collected from pigs on day 0 (prior to challenge) and on days 3, 7, 10, and 14 of challenge. Overall, baseline cytokine levels as determined on day 0, considered a baseline, were similar between ΔSRCR5 and wild-type pigs. However, the monokine induced by gamma interferon (IFN-γ) (MIG; also known as CXCL9) was found to show consistently higher levels in wild-type pigs until day 10, after which no significant difference was detected. MIG is a T-cell chemoattractant to inflammation sites and involved in the repair of tissue damage. In wild-type animals, MIG was strongly upregulated on days 7 and 10 of the challenge (36) (Fig. 4H). The level of chemokine ligand 3-like 1 (CCL3L1) (an isoform of macrophage inflammatory protein 1α [MIP-1α]), involved in the inflammation response via CCR5 signaling and downregulated by IL-10 (37), was found to be higher in wild-type than in ΔSRCR5 animals (Fig. 4J). As IL-10 levels were found to be comparable in both genotype groups, IL-10-mediated downregulation is unlikely to be the cause of low CCL3L1 levels. In wild-type animals, the level of CCL3L1 was elevated in the serum on days 10 and 14, whereas no significant IL-10 elevation was found to occur over the period of the challenge (Fig. 4O).

We observed sequential cytokine responses to PRRSV-1 infection in wild-type animals, with an early increase in the levels of IFN-α, IL-17A, and the IL-1 receptor antagonist (IL-1ra) (Fig. 4A to C), followed by an increase in the levels of IL-4, IL-6, and IL-8 at the high point of viremia, from 7 days postinoculation (dpi) (Fig. 4D to F). Increased levels of MIG and MIP-1β (also known as CCL4) were observed only transiently at 10 dpi (Fig. 4G and H). In the last period of the challenge, with moderate but reducing levels of viremia, elevations in the levels of CCL3L1, granulocyte-macrophage colony-stimulating factor (GM-CSF), IL-12, and IL-1β were detected (Fig. 4I to M). All of these cytokine responses were restricted to wild-type animals, with no cytokine response being recorded for ΔSRCR5 pigs. IL-10, transforming growth factor β1 (TGFβ1), and IFN-γ levels showed no significant difference between wild-type and ΔSRCR5 pigs at any of the time points but were found to change significantly over time in the wild-type animals (calculated using two-way analysis of variance [ANOVA] and Sidak's multiple-comparison test) (Fig. 4N to P). IL-18 levels decreased significantly over time in wild-type animals but were not significantly different from those of ΔSRCR5 pigs at each time point (Fig. 4Q). The level of platelet endothelial cell adhesion molecule 1 (PECAM1) was significantly elevated on day 3 of the challenge and decreased on day 10 compared to the levels in ΔSRCR5 pigs (Fig. 4R). No significant difference in the levels of IL-1α and IL-13 was found between ΔSRCR5 and wild-type pigs or over time (Fig. 4S and T).

## DISCUSSION

The results of this study show that ΔSRCR5 pigs are healthy under standard husbandry conditions and maintain the biological function of the CD163 protein while being resistant to PRRSV infection. So far, we have bred three generations of edited animals with over 10 litters and have not observed any abnormalities in breeding.

ΔSRCR5 pigs were generated, as previously described (15), by using two short guide RNAs (sgRNAs) flanking exon 7 of *CD163* CRISPR/Cas9. Here, we have shown that heterozygous and homozygous animals can be bred naturally and yield normal-litter-size offspring. Our previous data showed that primary PAMs and peripheral blood monocyte (PBMC)-derived macrophages from ΔSRCR5 pigs are fully resistant to PRRSV-1 subtype 1, 2, and 3 infection as well as to both typical and atypical PRRSV-2 (15). To confirm that these results translated to the *in vivo* model, we show here that ΔSRCR5 pigs are completely resistant to infection with a highly virulent PRRSV-1 subtype 2 strain. The edited animals displayed no clinical or pathological signs of infection, no viral replication was observed, and no cytokine response (indicative of low-level virus replication) was observed. This confirms that our previous *in vitro* results directly translate to the *in vivo* situation.

The ΔSRCR5 CD163 protein was previously detected on the surface of CD163-expressing macrophages using a native-confirmation antibody (15). Analysis of the ΔSRCR5 CD163 amino acid sequence *in silico* using RaptorX predicts that posttranslational folding will yield a protein that closely mimics the structure of full-length CD163. The expression of ΔSRCR5 CD163 in animals has several advantages over previously described PRRSV-resistant CD163 knockout animals generated by the random introduction of a premature stop codon in exon 3 or exon 7 of the CD163 gene (14, 23). Free hemoglobin, often released following hemolytic events, can cause serious toxicity to a system (reviewed in reference 38). CD163 is a direct mediator of Hb-Hp complex uptake into macrophages, which sequesters and degrades this potentially toxic compound (39). We have previously shown that PBMC-derived macrophages from ΔSRCR5 animals are still capable of CD163-mediated Hb-Hp uptake, as demonstrated by HO-1 upregulation and the uptake of the fluorescently labeled Hb-Hp complex (Hb_AF488_-Hp) (15). All ΔSRCR5 animals showed normal hemoglobin levels in their blood, confirming the proper clearance of Hb-Hp complexes. Yang et al. recently remade pigs with a premature stop codon in exon 7 of CD163, resulting in a functional CD163 knockout, as previously reported (40). Surprisingly, that paper claims that PBMC-derived macrophages from CD163 knockout animals are able to take up Hb_AF488_-Hp complexes *in vitro* (23), a result that directly contradicts the findings by Schaer et al. and Nielsen et al. (39, 41) in human macrophages, Etzerodt et al. in cells with murine CD163 (42), and Boretti et al. in canine macrophages (49), all of which highlight the essential requirement for CD163 to be present for Hb-Hp uptake in macrophages.

CD163, in both its cell-bound as well as its secreted forms, has been shown to have multiple other functions in addition to Hb-Hp scavenging (reviewed in reference 18). One important aspect is the regulatory function of soluble CD163 following inflammation and ischemic repair, which was found to result in enhanced regeneration activity in CD163 knockout mice, resulting in abnormal peripheral blood vessel development and systemic rather than local regeneration after injury (43). Soluble CD163 is also able to bind Staphylococcus aureus, which promotes the recognition, phagocytosis, and killing of this important livestock and human pathogen (44). Soluble CD163 is not the result of alternative splicing but results from proteolytic cleavage, likely by the metalloprotease ADAM17 in the juxtamembrane area following SRCR domain 9 of the protein (45). Proper folding of CD163 and accessibility of this area are essential for the secretion of soluble CD163. Here, we show the presence of soluble CD163 in the serum of ΔSRCR5 pigs at levels comparable to those in wild-type animals.

CD163 knockout mice have been reported to be significantly more susceptible to intra-abdominal sepsis, which is linked to haptoglobin-HMGB1 signaling and the cytokine response (46). It was also found that CD163 plays a role in asthmatic human patients, and CD163 knockout mice were found to have increased airway eosinophils and mucus cell metaplasia linked to CCL24 chemokine signaling upon dust mite challenge (47). We analyzed the function of ΔSRCR5 CD163 in signaling and the cytokine response by measuring the baseline cytokine levels in ΔSRCR5 pigs compared to their wild-type counterparts. We also monitored the cytokine levels during the course of the *in vivo* PRRSV challenge to identify any changes that could result from low-level PRRSV replication. Whereas we found an orchestrated sequence of inflammation and immune response signaling in the PRRSV-infected wild-type animals, no cytokine response was observed in the ΔSRCR5 pigs. Of the panel of 20 cytokines analyzed, the levels of only one cytokine, CCL3L1, were significantly different between the two groups of animals over the entire course of the challenge. The inflammation response protein CCL3L1 is involved in the inflammation response and is downregulated by IL-10, but no significant differences in IL-10 levels between the two genotype groups could be found. We have no explanation for the higher CCL3L1 level in ΔSRCR5 pigs, but the limited time frame and number of animals in this study warrant further investigation of this cytokine in the future. Another cytokine, MIG, showed higher levels in wild-type animals up to day 10, but no significant difference was observed on day 14. It will be interesting to investigate this observation further, over a longer period and with larger numbers of animals.

The creation of ΔSRCR5 pigs holds tremendous opportunity for the pork industry worldwide to improve both animal welfare and productivity at the same time. PRRSV infection has immunomodulatory outcomes and plays an important role in polymicrobial disease, such as the porcine respiratory disease complex. As such, PRRSV-resistant animals could benefit general health and decrease the need for antimicrobial use at the same time. However, for the implementation of next-generation breeding/genome-editing techniques in animal production, both consumer acceptance and the legislative framework need to be in place.

## MATERIALS AND METHODS

All animal work was approved by The Roslin Institute's and the University of Edinburgh's Protocols and Ethics Committees as well as the ethics group at Moredun Scientific Ltd. The experiments were carried out under the authority of UK Home Office project licenses PPL60/4518, PPL60/4482, and PPL70/8827 under the regulations of the Animal (Scientific Procedures) Act of 1986. Humane endpoints were clearly defined.

### Cells and viruses.

Primary pulmonary alveolar macrophages (PAMs) for the propagation of PRRSV-1 subtype 2 strain BOR-57 (isolated from a sample from a Belarusian pig in 2009 by T. Stadejek) were harvested from wild-type research animals aged 6 to 9 weeks as previously described (48). Briefly, animals were euthanized according to a schedule I method. Lungs were removed and transferred on ice to a sterile environment. Lung lavage with warm phosphate-buffered saline (PBS) and gentle massage were used to recover PAMs. Cells were collected by centrifugation for 10 min at 400 × *g*. When necessary, red blood cells were removed using red cell lysis buffer (10 mM KHCO_3_, 155 mM NH_4_Cl, 0.1 mM EDTA [pH 8.0]) for 5 min before washing again with PBS. Cells were collected by centrifugation as described above and frozen in a solution containing 90% fetal bovine serum (FBS) (heat inactivated [HI]; GE Healthcare) and 10% dimethyl sulfoxide (DMSO) (Sigma). Cells were frozen gradually at 1°C/min in a −80°C freezer before being transferred to −150°C.

PAM cells were cultivated in a solution containing RPMI 1640, GlutaMAX (Invitrogen), 10% FBS (HI; Gibco), 100 IU/ml penicillin, and 100 μg/ml streptomycin (Invitrogen) (RPMI^+/+^).

### Breeding and genotyping of animals.

Unrelated founder animals generated by zygote injection of Cas9 mRNA and sgRNAs SL26 and SL28, as described previously (15), were bred to each other or to wild-type animals to generate heterozygous F1 offspring. F1 animals with a double-strand break and religation without insertions or deletions at the cutting sites of SL26 and SL28 were selected for breeding the F2 generation. Animals were genotyped as described previously (15); briefly, genomic DNA was extracted from ear biopsy specimens using the DNeasy blood and tissue kit (Qiagen). The region spanning intron 6 to exon 8 was amplified using primers oSL46 (ACCTTGATGATTGCGCTCTT) and oSL47 (TGTCCCAGTGAGAGTTGCAG), generating a 904-bp product from the intact allele and a 454-bp product if the complete deletion of exon 7 had occurred. The PCR products were analyzed by separation on a 1% agarose gel and by Sanger sequencing.

### Animal challenge with BOR-57.

Four days prior to transfer of the animals to the specific-pathogen-free unit (SPFU), blood and serum samples were taken from all animals by jugular venipuncture, and the blood samples were subjected to whole-blood-count analyses. Sera were screened by using the Idexx PRRSV X3 ELISA to confirm that none of the animals had previously been exposed to PRRSV. Animals were acclimated in the SPFU for 1 week prior to challenge.

Infectivity of BOR-57 stocks was assessed using a TCID_50_ assay on PAMs immediately after production, prior to challenge, and on both challenge dates after administration. The BOR-57 inoculum was tested for the absence of mycoplasma and other major viral pathogens. Animals were inoculated intranasally in the left nostril with 5 ml of 1E6 TCID_50_/ml BOR-57 in RPMI^+/+^. Body weights of the animals were measured on day 0 prior to challenge and on day 7 and day 14 prior to euthanasia. Serum samples were collected on day 0 prior to challenge and on days 3, 7, 10, and 14 prior to euthanasia by jugular venipuncture into Vacutainer tubes. After clotting, samples were centrifuged at 2,000 × *g* for 10 min at 4°C, and samples were aliquoted and frozen at −80°C for further analysis. Clinical observations were recorded daily, making note of the rectal temperature, demeanor, nasal discharge, coughing, and respiration. Feeding, water consumption, and general health were observed and recorded daily. Humane endpoints were defined prior to challenge. No animal reached the criteria for premature termination during the challenge.

### Necropsy, histopathology, and immunohistochemistry.

On day 14 of the challenge, animals were euthanized according to a schedule I method. During necropsy, the lungs were removed and initially assessed, and detailed photographs were taken from the dorsal and ventral sides for detailed scoring of macroscopic lung lesions. An established scoring system, based on the approximate contribution of each lung section to the complete lung volume, was employed as previously described (29). Briefly, each lung lobe is assigned a number to reflect the approximate volume percentage of the entire lung represented. The affected area of each lobe is scored relative to the assigned volume percentage. Lung, mediastinal lymph node, and PAM samples were collected and frozen, and lung and lymph node samples were fixed in 10% neutral buffered formalin. Formalin-fixed sections were embedded in paraffin and routinely processed for histological examination with hematoxylin and eosin staining. Lung sections were scored for the presence and severity of interstitial pneumonia, ranging from 0 (normal) to 6 (severe diffuse), as previously described (29). Immunohistochemical analysis of lung and lymph nodes for the detection of PRRSV antigen was performed as previously described (30), using a mixture of two monoclonal antibodies, SDOW-17 at 1:5,000 and SR-30 at 1:1,500 (both Research|Technology|Innovation [RTI]), as primary antibodies. Sections were counterstained with hematoxylin.

### Assessment of PRRSV and anti-PRRSV antibody levels in serum.

Viral RNA was extracted from the sera collected on days 0, 3, 7, 10, and 14 using the QIAamp viral RNA minikit (Qiagen) according to the manufacturer's instructions. Viral RNA levels were assessed by reverse transcription-quantitative PCR (RT-qPCR) using the GoTaq 1-Step RT-qPCR system (Promega) and analyzed on a LightCycler II 480 instrument (Roche). Viral RNA extracted from the PAM cell culture supernatant with a known multiplicity of infection (MOI) and a custom-synthesized DNA fragment (Invitrogen) of a known concentration (GAGAGCGGCCGCTAATACGACTCACTATAGTCAGCTGTGTCAGCTGCTGGGAAAAATGATGAAATCCCAGCGCCAGCAACCCAGGGGAGGACAGGCCAACAAAAGGAAAAAGCCTGAGAAGCCTCATTTTCCCTTGGCTGCTGAAGATGACGTTCGGCACCATCTCACCGCAACTGAGCGTTCCCTCTGTCTGCAATCGATCCAGACAGCCTTCAATCAGGGTGCAGGAACTGCGTCGCTTTCACCCAGTGGGAAGGTCAGTTTTCAGGTAGAGTTCATGCTGCCCCTGCAGGGAGA) were used as standards. The primers used were BOR57_FWD (GAAATCCCAGCGCCAGCAAC) and BOR57_REV (TTCCCACTGGGTGAAAGCGA).

### Assessment of soluble CD163 serum levels.

Serum samples collected a week prior to and on day 0 of the challenge were analyzed for the presence and level of soluble CD163. A sandwich ELISA was performed using the porcine CD163 ELISA kit (Elabscience) according to the manufacturer's instructions and using serial dilutions of serum.

### Analysis of serum cytokine levels using cytokine arrays.

Serum samples collected on day 0 (prior to challenge) and on days 3, 7, 10, and 14 of the challenge were analyzed for the levels of 20 different cytokines. Cytokine array analyses were performed using porcine cytokine antibody array A (catalog number ab197479) and array B (catalog number ab197480) (both from Abcam) according to the manufacturer's instructions.
