# Erratum for Burkard et al., “Pigs Lacking the Scavenger Receptor Cysteine-Rich Domain 5 of CD163 Are Resistant to Porcine Reproductive and Respiratory Syndrome Virus 1 Infection”

**DOI:** 10.1128/JVI.00951-20

**Published:** 2020-07-16

**Authors:** Christine Burkard, Tanja Opriessnig, Alan J. Mileham, Tomasz Stadejek, Tahar Ait-Ali, Simon G. Lillico, C. Bruce A. Whitelaw, Alan L. Archibald

**Affiliations:** a The Roslin Institute, Royal (Dick) School of Veterinary Studies, University of Edinburgh, Easter Bush, Midlothian, United Kingdom; b Department of Veterinary Diagnostic and Production Animal Medicine, College of Veterinary Medicine, Iowa State University, Ames, Iowa, USA; c Genus plc, DeForest, Wisconsin, USA; d Warsaw University of Life Sciences, Faculty of Veterinary Medicine, Department of Pathology and Veterinary Diagnostics, Warsaw, Poland

## ERRATUM

Volume 92, no. 16, 2018, https://doi.org/10.1128/JVI.00415-18. Page 1: The author affiliation line should appear as shown here. The spelling of “Roslin” has been corrected.

